# A Case Report of a Rare, but Important, Cause of Delerium Presenting to an Emergency Department

**DOI:** 10.5811/cpcem.31059

**Published:** 2025-02-26

**Authors:** Daniel G. Miller

**Affiliations:** University of Iowa Healthcare, Departments of Emergency and Internal Medicine, Iowa City, Iowa

**Keywords:** delayed post-hypoxic leukoencephalopathy, delayed neurologic sequelae, neuroimaging, opiate overdose, opioid overdose

## Abstract

**Introduction:**

Delayed post-hypoxic leukencephalopathy is a rare cause of acute neuropsychiatric decline diagnosable in the emergency department (ED), but it has not been described in the emergency medical literature. We present a case report of a pathognomonic presentation.

**Case Report:**

A 53-year-old man developed akinetic mutism 14 days after being discharged from a hospitalization for fentanyl overdose. Magnetic resonance imaging demonstrated symmetric frontoparietal white matter demyelination.

**Conclusion:**

Delayed post-hypoxic leukencephalopathy can present to the ED as altered mental status days to weeks after apparent full recovery from an initial episode of cerebral hypoxia. This report will help emergency physicians avoid missing this diagnosis.

## INTRODUCTION

Delayed post-hypoxic leukencephalopathy (DPHL) is a demyelinating brain disorder that causes an abrupt onset of neuropsychiatric dysfunction (either acute onset of parkinsonism or akinetic mutism) days or weeks after apparent full recovery from coma following an episode of profound cerebral hypo-oxygenation. This biphasic presentation distinguishes DPHL from acute anoxic encephalopathy.^1^ The lucid interval (median 22 days) makes the emergency department (ED) a likely venue for presentation.^2^ Characteristic findings on magnetic resonance imaging (MRI) aid in diagnosis. The increasing use of MRI in EDs will give emergency physicians (EP) increasing opportunities to make (or to miss) the diagnosis of DPHL.^3^,^4^ We present a case report of a patient presenting with acute akinetic mutism three weeks after appearing to fully recover from a fentanyl overdose. His presentation demonstrates characteristic historical, physical, and imaging features of DPHL.

## CASE REPORT

A 53-year-old man with no known neurologic or psychiatric history was brought to the ED after being found unresponsive after accidental fentanyl overdose. Emergency medical services initially noted apnea and percentage oxygen saturation in the 40s. After administration of naloxone and oxygen he was observed to be moving all four extremities but remained unresponsive to verbal stimuli in the ED; so, he was intubated for airway protection. Fever and hypotension prompted initiation of sepsis care. Head computed tomography (CT) showed no acute findings. A urine toxicology panel was positive for fentanyl, amphetamines, and benzodiazepines, but negative for opiates. Over the following day his vital signs stabilized, and on day three an electroencephalogram (EEG) showed no signs of epileptiform activity. He tolerated extubation and had no focal neurologic deficits. Seven days after admission he was discharged to home and resumed work.

**Image 1:**
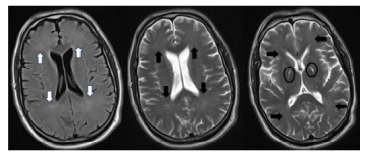
Magnetic resonance imaging at onset of neuropsychiatric decline (19 days after initial visit) showing symmetric subtle hyperintensity in frontal and parietal lobes (axial FLAIR sequence on far left with white arrows, axial T2 sequence in middle and far right with black arrows) and hyperintensity of the bilateral globi palidi (ovals) with normal-appearing gray matter.

Nineteen days after the anoxic event, family members found the patient “spaced out and drowsy” and brought him to the ED. He had normal vital signs, but speech was very slow, nearly mute, and he answered orientation questions with “I am here.” Movement was markedly slow. He would squeeze fingers placed in his palms, but he did not follow any complex commands and was incontinent of urine. Illicit drug toxicity, hypercarbia, electrolyte abnormalities, traumatic brain injury, and meningitis were all considered and were evaluated with the following studies: toxicology screening was negative, venous blood gas and chemistry profiles were normal, CT brain showed signal hyperintensity in the basal ganglia but no other acute finding, and lumbar puncture yielded clear fluid with normal cell counts and no organisms or viruses detected. An MRI (Image 1) showed hyperintensity of basal ganglia and hyperintensity of white matter tracks. This raised concern for an autoimmune encephalopathy and for opioid-related conditions that cause encephalopathy persisting long after the offending drug has been metabolized: DPHL and opioid-induced leukencephalopathy (OIL).

During inpatient care methylprednisolone 1gram was administered daily for potential autoimmune process, and plasmapheresis was initiated. He later became tremulous and diaphoretic with tachycardia, hypertension, and muscle stiffening and was intubated for airway protection and given valproate for possible seizure disorder. Subsequent EEG showed no ictal changes yet did show diffuse delta slowing consistent with encephalopathy, but not seizure. We considered neuroleptic malignant syndrome (NMS) and serotonin syndrome (SS), as well as tetanus and strychnine poisoning as causes of this change. The patient had received haloperidol early in his course, although only 2 milligrams total. As he was without fever at that time, and he had no clonus or hypereflexia, NMS and SS were unlikely. Additionally, his neurocognitive symptoms continued for several days after resolution of this acute change and without the administration of any further serotonergic or anti-dopaminergic agents, ruling out NMS and SS as causes of his ongoing inability to follow commands. Strychnine poisoning and tetanus cause muscular hyperactivity in awake patients, but our patient had depressed mental status throughout his hospital stay. While his hypertonicity began several days into his hospital course and resolved within minutes, strychnine poisoning is rapid in onset, and tetanus causes more prolonged symptoms.

**CPC-EM Capsule**

What do we already know about this clinical entity?

*Cerebral hypoxia can cause lasting neurologic sequelae, but emergency physicians (EP) may be unaware of delayed post-hypoxic leukencephalopathy (DPHL)*.

What makes this presentation of disease reportable?

*This demonstrative emergency department (ED) case of DPHL elucidates this lesser-known cause of acutely altered mental status*.

What is the major learning point?

*When a patient presents with new-onset parkinsonism or akinetic mutism days to weeks after recovering from a profound hypoxic episode, DPHL should be considered*.

How might this improve emergency medicine practice?

*This case report will help EPs recognize DPHL, a condition that is likely to become increasingly diagnosable in the ED*.

**Image 2:**
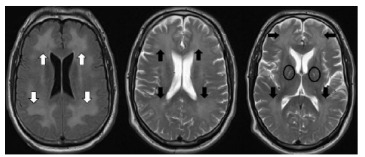
Magnetic resonance imaging two days after onset of neuropsychiatric decline (20 days after initial visit) showing more apparent symmetric hyperintensity in frontal and parietal lobes (axial FLAIR sequence on far left with white arrows, axial T2 sequence in middle and far right with black arrows) and hyperintensity of the bilateral globi palidi (ovals) with otherwise normal-appearing gray matter.

A repeat MRI (Image 2) showed progression of symmetric hyperintensity in frontal and parietal lobes and bilateral globi palidi hyperintensity otherwise sparing the gray matter; posterior circulation areas were spared.

We considered posterior reversible encephalopathy syndrome (PRESS); however, his blood pressure was within normal limits upon presentation of encephalopathy, and his condition did not improve with supportive care. Additionally, while PRESS tends to affect the cortical regions and predominates in the posterior areas of the brain, our patient’s abnormal MRI findings were seen in the subcortical regions of the frontal and parietal areas.

**Image 3:**
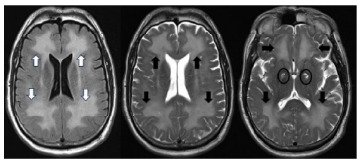
Magnetic resonance imaging 20 days after onset of neuropsychiatric decline (30 days after initial visit) showing marked progression of symmetric hyperintensity in frontal and parietal lobes (axial FLAIR sequence on far left with white arrows, axial T2 sequence in middle and far right with black arrows) and hyperintensity of the bilateral globi palidi (ovals) with normal-appearing gray matter.

Both OIL and DPHL cause mutism, incontinence, confusion, and extrapyramidal symptoms. And both cause symmetric changes that predominantly affect white rather than gray matter, suggesting that myelinotoxicity is a unifying feature. In our case the biphasic presentation distinguished DPHL from OIL, in which a lucid interval after the inciting hypoxic event is not observed.^5^ Thirty days after his hypoxic episode an MRI (Image 3) demonstrated marked symmetric white matter signal hyperintensity in the frontal and parietal lobes with necrosis of the medial globi pallidi.

It was at this time that we considered the diagnosis DPHL to be conclusive and, given his progressive symptoms, the likelihood of recovery to be extremely low. Palliative care met with the family, and after discussions of prognosis and the patient’s known values his care we transitioned to comfort-focused care. After extubation he had spontaneous eye-opening during which time he appeared to stare at nothing and tracked neither examiners nor family members. He did not speak, follow commands, or eat when offered food. He died peacefully four days later.

## DISCUSSION

Both OIL and DPHL cause abrupt neuropsychiatric decline and appear to be due to myelin disruption. Opioid-induced leukencephalopathy has commonly been described after inhalation of vaporized heroin (“chasing the dragon”) but has also been described after other methods of heroin use and after use of oxycodone. In DPHL an acute neuropsychiatric decline follows an apparent full recovery from a period of profound cerebral hypoxia. It was first described in 1936 as a sequelae of carbon monoxide (CO) toxicity and has been observed after cardiac arrest, asphyxia, drug overdose, and strangulation.^6^–^8^ Common to all causes of DPHL is a period of profound cerebral anoxia. Delayed post-hypoxic leukencephalopathy and OIL have significant clinical overlap, but there are some features to differentiate the two. As mentioned above, DPHL is characteristically biphasic while OIL is not. Whereas the neuroimaging findings in DPHL typically involve the white matter of the frontal and parietal regions, those in OIL most commonly affect the cerebellum, the posterior cerebrum, and the posterior limbs of the internal capsule.^9^

The decline in DPHL is characterized by symptoms that fit one or both of two categories: parkinsonism and akinetic mutism.^10^ Common parkinsonian symptoms are rigidity, tremor, and hallucinations. Case reports are mixed on the role of levodopa to treat DPHL-related parkinsonism; however, caution around use of antidopaminergic agents seems prudent, as these could worsen symptoms. Akinetic mutism symptoms include apathy, staring, markedly slowed speech, and functional incontinence. Presentations can overlap with both types, and common physical exam findings include frontal release signs, upper motor neuron signs, and primitive responses to noxious stimuli. This is consistent with our patient’s grasping reflexes, inanition, and periods of increased tone. Several features are suggestive that myelinotoxicity suffered during the hypoxic event leads to DPHL: The cerebral white matter is affected, while gray matter is spared, and elevation of myelin basic protein in cerebral spinal fluid is strongly associated with DPHL.^11^ The delayed onset of symptoms correlates with the pattern of myelin secretion occurring on average every 20 days.^12^

In most cases, CT will not be diagnostic, and MRI will be required for diagnosis. The following findings are pathognomonic: cerebral white matter hyperintensities seen on T2-weighted images that symmetrically affect the dorsal frontal and parietal lobes while sparing the cortex, cerebellum, and brainstem.^13^ On diffusion-weighted sequences a diffusion restriction involving the globi pallidi, which are traversed by numerous myelinated axons, can show high diffusion restriction, indicating cytotoxic edema.^14^ All these features were present in our case. These findings help distinguish DPHL from other diagnoses in the differential.

Steroids and plasmapheresis have been attempted for treatment of DPHL but have not been reported to be of benefit. Zolipidem has been reported to transiently improve alertness and orientation in patients after anoxic brain injury who are no longer comatose.^15^ We did not observe any improvement when we administered it to our patient. Care for DPHL is focused on support and rehabilitation if the patient can participate. Among patients with CO-associated DPHL the likelihood of recovery has an inverse relationship to age at onset. The majority of those who survived hospitalization recovered over three to six (median four) months and suffered continued difficulties related to frontal lobe function.^9^ For patients who progress to coma during the second phase, recovery from DPHL has not been reported.

## CONCLUSION

Delayed post-hypoxic leukencephalopathy is a cause of altered mental status that EPs should consider in patients who present with acute neuropsychiatric decline days to weeks after recovering from a severe episode of cerebral hypoxia. Characteristic symptoms are new-onset parkinsonism or akinetic mutism. If an MRI is performed, specifically look at the T2 sequences for the pathognomonic findings of symmetric, white matter hyperintensity involving the anterior portion of the cerebrum, while sparing the areas supplied by posterior circulation.

## References

[1] Messina Z, Hays Shapshak A, Mills R (2024). Anoxic encephalopathy. StatPearls.

[2] Choi IS (1983). Delayed neurologic sequelae in carbon monoxide intoxication. Arch Neurol.

[3] Sánchez Y, Yun BJ, Prabhakar AM (2017). Magnetic resonance imaging utilization in an emergency department observation unit. West J Emerg Med.

[4] Baloescu C (2018). Diagnostic imaging in emergency medicine: How much is too much?. Ann Emerg Med.

[5] Min SK (1986). A brain syndrome associated with delayed neuropsychiatric sequelae following acute carbon monoxide intoxication. Acta Psychiatr Scand.

[6] Shillito F, Drinker C, Shaughnessy T (1936). The problem of nervous and mental sequelae in carbon monoxide poisoning. JAMA.

[7] Hori A, Hirose G, Kataoka S (1991). Delayed postanoxic encephalopathy after strangulation. serial neuroradiological and neurochemical studies. Arch Neurol.

[8] Protass LM (1971). Delayed postanoxic encephalopathy after heroin use. Ann Intern Med.

[9] Offiah C, Hall E (2008). Heroin-induced leukoencephalopathy: characterization using MRI, diffusion-weighted imaging, and MR spectroscopy. Clin Radiol.

[10] Shprecher D, Mehta L (2010). The syndrome of delayed post-hypoxic leukoencephalopathy. NeuroRehabilitation.

[11] Shprecher DR, Flanigan KM, Smith AG (2008). Clinical and diagnostic features of delayed hypoxic leukoencephalopathy. J Neuropsychiatry Clin Neurosci.

[12] Zamora CA, Nauen D, Hynecek R (2015). Delayed posthypoxic leukoencephalopathy: a case series and review of the literature. Brain Behav.

[13] Beeskow AB, Oberstadt M, Saur D (2018). Delayed post-hypoxic leukoencephalopathy (DPHL): an uncommon variant of hypoxic brain damage in adults. Front Neurol.

[14] Chu K, Jung KH, Kim HJ (2004). Diffusion-weighted MRI and 99mTc-HMPAO SPECT in delayed relapsing type of carbon monoxide poisoning: evidence of delayed cytotoxic edema. Eur Neurol.

[15] Shames JL, Ring H (2008). Transient reversal of anoxic brain injury-related minimally conscious state after zolpidem administration: a case report. Arch Phys Med Rehabil.

